# Major Ozonated Autoheamotherapy Alleviates Skeletal Muscle Ischemia/Reperfusion Injury by Regulating Nrf2/HO‐1 Pathway

**DOI:** 10.1002/kjm2.70039

**Published:** 2025-05-23

**Authors:** Hui‐Zhuang Guo, Sheng‐Long Yu, Han‐Wei Chen

**Affiliations:** ^1^ The First Affiliated Hospital of Jinan University Guangzhou China; ^2^ Department of Radiology, The Affiliated Panyu Central Hospital Guangzhou Medical University Guangzhou China; ^3^ Department of Cardiovascular, The Affiliated Panyu Central Hospital Guangzhou Medical University Guangzhou China; ^4^ Panyu Health Management Center (Panyu Rehabilitation Hospital) Guangzhou China

**Keywords:** ischemia/reperfusion, major ozonated autoheamotherapy, Nrf2/HO‐1, oxidative damage

## Abstract

Skeletal muscle ischaemia/reperfusion (I/R) injury remains a clinically significant condition characterized by muscular dystrophy. Although ozone therapy has shown protective potential against I/R injury in animal models of various organs including skeletal muscle, its precise mechanistic underpinnings require further elucidation. This investigation evaluates the therapeutic potential of major ozonated autohemotherapy (MOAH) for skeletal muscle I/R injury management. Utilizing a rat femoral artery ligation/release model, we demonstrated that MOAH pretreatment substantially alleviated histopathological damage through hematoxylin–eosin/Masson staining analyzes, diminished skeletal muscle apoptosis via terminal deoxynucleotidyl transferase dUTP nick‐end labeling and Western blot, and reduced tissue edema as quantified by wet weight ratios. Serum biomarker assessments confirmed decreased creatine kinase and lactate dehydrogenase levels with MOAH administration. In oxygen–glucose deprivation/reoxygenation (OGD/R)‐treated L6 myoblast models, ozone pretreatment enhanced cellular proliferation capacity while attenuating apoptosis and mitochondrial dysfunction. Subsequent analyzes revealed ozone's regulatory effects on oxidative stress markers (MDA content, SOD and CAT activity) and inflammatory factors (TNF‐α and IL‐1β) across both in vivo and in vitro models. Mechanistic evaluations through Western blot and reverse transcription quantitative real‐time polymerase chain reaction techniques identified MOAH‐induced activation of the Nrf2/HO‐1 signaling pathway, with observed abolition of protective efficacy under Nrf2 knockdown conditions. These results collectively establish that MOAH mitigates skeletal muscle I/R injury through Nrf2/HO‐1 pathway modulation, providing substantive mechanistic justification for its clinical implementation in I/R injury therapeutics.

## 1 Introduction

Peripheral artery disease (PAD) is caused by atherosclerosis, resulting in poorly perfused peripheral vessels, which leads to ischemia and hypoxia that can cause disability and death [1, 2]. As one of the most serious forms of PAD, acute limb ischemia (ALI) progresses acutely and rapidly, leading to amputation and death if not treated properly [3, 4]. Ischemia typically results in energy depletion, toxic metabolite buildup, increased tissue acidity, and activation of phospholipases and lysozymes, causing cellular damage. This damage is further intensified by reperfusion, compromising cell viability and ischemic organ function, a phenomenon known as ischemia/reperfusion (I/R) injury [5]. The high metabolic activity of skeletal muscle makes it highly vulnerable to I/R injury. Skeletal muscle I/R impacts both the viability and function of the affected tissues and can trigger systemic inflammatory response syndrome, potentially resulting in multi‐organ dysfunction syndrome in severe cases [6, 7]. Skeletal muscle I/R injuries are currently treated ineffectively, making it urgent to find an effective and feasible treatment method.

Skeletal muscle I/R pathogenesis is mainly related to inflammatory response, oxidative stress, and mitochondrial dysfunction [8]. Medical ozone is a gas mixture composed of ozone (O_3_) and oxygen (O_2_) that has been proven effective for treating a wide variety of diseases because of its antioxidant, immunomodulatory, and microcirculatory effects [9, 10]. In clinical practice, ozone therapy can be administered in many ways, including hydrotherapy, oil therapy, and autohemotherapy. Major ozonated autoheamotherapy (MOAH) i.e., slow re‐infusion of autologous blood and O_2_/O_3_ mixtures, is one of the most reliable and advanced forms [11]. MOAH has shown positive therapeutic effects in acute cerebral infarction [12], cerebral ischemia [13], acute herpes zoster [14], insomnia with myofascial pain syndrome [15], and peripheral arterial occlusive disease [16]. Moreover, MOAH protects against renal I/R injury, possibly as a result of anti‐inflammatory effects and oxidative stress reduction [17]. Ozone could alleviate I/R injury in rat skeletal muscle by reducing oxidative and nitrosative stress parameters and increasing antioxidant enzyme levels [18]. Nevertheless, current understanding of MOAH's therapeutic mechanisms in skeletal muscle I/R injury remains limited, with few studies systematically investigating its biological effects and molecular pathways.

Therefore, this study intends to establish models of skeletal muscle I/R injury and to investigate the protective effect of MOAH on skeletal muscle I/R injury.

## 2 Materials and Methods

### 2.1 Experimental Animals

Seventy healthy 6–8 weeks old male specific pathogen free (SPF) grade Sprague Dawley (SD) rats, weighing 160‐180 g, were provided by Chengdu DOSSY EXPERIMENTAL Animal Co., LTD (Sichuan, China). They were housed in separate cages, maintained on a free diet and water (22°C ± 2°C, relative humidity of 40%–60%, and 12 h light cycle). Acclimatization feeding was conducted for 7 days. All procedures were approved by the Ethics Committee (PY‐LL‐2024‐0128) and followed the ARRIVE guidelines.

### 2.2 Animal Modeling and Grouping

I/R model was established following earlier descriptions, with minor modifications [19, 20]: The rats were put under anesthesia using 2% pentobarbital sodium at 40 mg/kg via intraperitoneal injection and laid on their backs. The right lower abdomen and groin were shaved, and a transverse incision was made in the groin to expose the right inguinal blood vessels. The right femoral vasculature (superficial and deep branches) underwent standardized occlusion using vascular clamps, with complementary circumferential ligation via an elastic rubber ring applied at the right inguinal region. Ischemic efficacy was confirmed through multimodal verification—observation of distal limb pallor, cyanosis, and temperature reduction, coupled with quantitative laser Doppler perfusion imaging demonstrating > 90% reduction in distal blood flow throughout the 4 h ischemic phase. After 4 h of ischemia, the vascular clamp was opened and the elastic rubber ring was cut off. The rats' toes and palms turned from dark purple to pink, restoring perfusion, and 24 h of reperfusion was induced.

Seventy SD rats were randomly divided into the 7 groups (*n* = 10): sham: rats undergoing surgical operation only without clamping of blood vessels and right lower limb ring ligation; I/R: rats modeled as described above; I/*R* + MOAH group: rats modeled after MOAH; I/*R* + Lv‐si‐NC: rats modeled after injection of Lv‐NC; I/*R* + Lv‐si‐Nrf2: rats modeled after Lv‐Nrf2 injection; I/*R* + MOAH + Lv‐si‐NC: rats modeled after MOAH and Lv‐si‐NC injection; I/*R* + MOAH + Lv‐si‐Nrf2: rats modeled after MOAH and Lv‐si‐Nrf2 injection.

MOAH was performed based on previous protocols [17, 21]: An O_2_/O_3_ gas mixture consisting of O_3_‐O_2_ in equal volumes was prepared using an ozone generator (Hermann, Germany). Caudal vein blood (2 mL per rat) was drawn using a heparinized syringe and exposed to the prepared gas mixture (2 mL). The sufficiently ozonated blood was slowly injected into rats through the caudal vein. MOAH was given once a day for 3 days before surgery. The Sham and I/R groups were treated with air following the same procedure as control.

Lentiviral vector construction: oe‐Nrf2 or si‐Nrf2 sequences targeting Nrf2 and corresponding negative controls were inserted into the pGC‐FU vector by genetic recombination. The vector and packaging plasmid (Genechem, Shanghai, China) were then co‐transfected into HEK293T cells. The supernatant containing the released lentiviral vector was collected, concentrated by ultracentrifugation, and adjusted to 2 × 10^8^ TU/mL. I/*R* + Lv‐oe‐NC, I/*R* + Lv‐oe‐Nrf2, I/*R* + MOAH + Lv‐si‐NC, and I/*R* + MOAH + Lv‐si‐Nrf2 groups were injected with the corresponding lentiviral vectors (10 μL/only) via tail vein 14 days pre‐I/R. The efficiency of Nrf2 knockdown or overexpression was confirmed through RT‐qPCR analysis and Western blot quantification. Lentiviral vector injections were administered according to the empirical validation in pilot experiments and aligned with well‐established methodologies from prior I/R investigations [22, 23].

### 2.3 Sampling

After 24 h of reperfusion, the rats were anesthetized, and the abdominal cavity was cut, and 2–3 mL of blood was taken from the inferior vena cava. The blood sample, treated with 3.8% sodium citrate for anticoagulation, underwent centrifugation at 3000 r/min for 10 min, followed by storage of the supernatant at −20°C.

Following 24 h of reperfusion, rats were humanely euthanized under deep anesthesia. The tibialis anterior muscle from the right hindlimb was surgically excised and immediately weighed using a precision analytical balance (wet weight measurement). Tissue samples underwent standardized dehydration in a calibrated oven maintained at 60°C for 72 h, followed by precise dry weight measurement. Quantitative assessment of tissue edema was performed using the gravimetric wet/dry (W/D) ratio calculation: (wet weight/dry weight) × 100%.

Fresh skeletal muscle specimens from the right hindlimb were collected and divided into two aliquots for parallel processing. The first subset underwent immediate flash‐freezing in liquid nitrogen and long‐term cryostorage at −80°C for subsequent biochemical analysis. The remaining tissue was fixed in 4% paraformaldehyde (24 h, 4°C), paraffin‐embedded using standard histology protocols, and sectioned at 5 μm thickness using a rotary microtome. Sections were mounted on poly‐L‐lysine‐coated slides for sequential histological analysis, including hematoxylin–eosin (H&E) staining, Masson's trichrome collagen visualization, and terminal deoxynucleotidyl transferase dUTP nick‐end labeling (TUNEL) apoptosis detection.

### 2.4 H&E Staining

Paraffin sections were routinely dehydrated in graded alcohol, permeabilized in xylene, and stained with hematoxylin solution for 3–5 min. Then, 1% hydrochloric alcohol was added for a 20 s reaction, followed by 1% ammonia for 30 s and 1% eosin solution for 5 min. Then, the sections were subjected to routine dehydration and clearance (80% ethanol for 5 min, 90% ethanol for 5 min, 95% ethanol for 5 min, absolute ethanol for 5 min, xylene for 10 min × 2 times). The sealed sections were observed microscopically. Muscle fiber disorganization and disappearance were used to score tissue damage, with a scale from 0 (normal) to 3 (severe). Inflammatory cell infiltration was also evaluated on the same scale [24].

### 2.5 Masson Staining

Following deparaffinization and rehydration, tissue sections underwent sequential staining with hematoxylin (5 min), acidic ethanol differentiation (1% HCl in 70% ethanol, 30 s), ponceau magenta solution (Absin, Shanghai, China, 8 min), phosphomolybdic acid treatment (5 min), and aniline blue counterstaining (10 min) prior to acetic acid differentiation. This optimized trichrome staining protocol yields distinct coloration: muscle fibers (red), collagen deposits (blue), and nuclei (deep gray). Collagen volume fraction (CVF, collagen area/total tissue area × 100%) quantification was performed using Image J software (Wayne Rasband, National Institutes of Health, USA) with the color deconvolution plugin, applying automated thresholding to isolate blue channel collagen signals.

### 2.6 TUNEL Staining

According to the TUNEL test kit (Keygene Biotech, Jiangsu, China), paraffin sections were routinely dehydrated, developed with DAB, and re‐stained with hematoxylin solution. After dehydration and permeabilization, the sections were sealed. TUNEL‐positive cells were defined as those with brown particles in the nucleus, i.e., apoptotic cells. The number of apoptotic cells and normal cells was analyzed by Image J software, and the apoptosis rate was calculated.

### 2.7 Measurement of Serum Indices

Rats were sacrificed after reperfusion, and serum samples were taken from the rats to measure creatine kinase (CK) and lactate dehydrogenase (LDH) levels using the kit (ELK Biotechnology, Wuhan, China).

### 2.8 Cell Culture and Treatment

Rat skeletal muscle myoblast L6 cell line National Collection of Authenticated Cell Cultures (Shanghai, China) was placed in a cell culture incubator (37°C, 5% CO_2_) with high glucose DMEM (Sigma‐Aldrich, USA) containing 10% fetal bovine serum and 1% penicillin–streptomycin. Cells in the logarithmic phase were digested with 0.25% trypsin.

#### 2.8.1 OGD/R Induction

Upon 80% confluence, the L6 cell culture medium was substituted with deoxygenated, glucose‐free DMEM (pH 7.4), followed by relocating the cells to a hypoxic chamber and perfusing them with 5% CO_2_ and 95% N_2_ for 12 h to trigger OGD/R. Subsequently, the cells underwent incubation in a standard medium and were maintained in standard culture conditions (5% CO_2_) for an additional 18 h to mimic reperfusion. The control cells underwent cultivation in a standard medium and were kept in typical conditions (5% CO_2_) for the same duration.

#### 2.8.2 Ozone Preparation

3 mL of DMEM was added to a centrifuge tube, 3 mL of ozone gas was collected from the ozone generating device into a sterile syringe, pumped into the DMEM, and oscillated with the DMEM for 5 min. Cells were cultured in the mixed medium during reperfusion. Cells were cultured separately with different concentrations (15, 30, 45, and 60 μg/mL) of ozone‐mixed medium, and cell viability was detected by the CCK8 method to select the optimal concentration of ozone.

#### 2.8.3 Cell Transfection

L6 cells were inoculated in 96‐well plates and transfected with oe‐NC, oe‐Nrf2, si‐NC, and si‐Nrf2 using Lipofectamine 2000 (Invitrogen, CA, USA). The efficiency of Nrf2 knockdown or overexpression was confirmed through RT‐qPCR analysis and Western blot quantification.

#### 2.8.4 Groups

Control group; OGD/R group; OGD/*R* + O_3_ group: cells cultured in the mixed medium (30 μg/mL O_3_) during reperfusion after OGD/R; OGD/*R* + oe‐NC group: cells transfected with oe‐NC and induced with OGD/R; OGD/*R* + oe‐Nrf2 group: cells transfected with oe‐Nrf2 and induced with OGD/R; OGD/*R* + O_3_ + si‐NC: cells transfected with si‐NC and cultured in the mixed medium (30 μg/mL O_3_) during reperfusion after OGD/R; OGD/*R* + O_3_ + si‐Nrf2: cells transfected with si‐Nrf2 and cultured in the mixed medium (30 μg/mL O_3_) during reperfusion after OGD/R.

### 2.9 CCK‐8 Assay

Cells underwent cultivation in 96‐well plates, with each well containing 100 μL or 5 × 10^3^ cells. After a 24‐h culture period, 10 μL of CCK‐8 solution (Sigma‐Aldrich) was introduced and left to incubate for 4 h. The measurement of optical density was taken at 450 nm using a microplate reader (Bio‐Rad, USA).

### 2.10 Flow Cytometry

Cells were introduced into 500 μL of 1 × binding buffer, combined with 5 μL of Annexin‐V‐FITC and 2.5 μL of PI, and analyzed using a flow cytometry (Becton, Dickinson and Company, USA). On the scatter plot, the outcomes displayed healthy live cells as FITC^−^/PI^−^ in the lower left quadrant, early apoptotic cells as FITC^+^/PI^−^ in the lower right quadrant, and late necrotic and apoptotic cells as FITC^+^/PI^+^. The apoptosis rate equals the sum of early apoptotic cells and the proportion of late apoptotic cells.

### 2.11 Mitochondrial Membrane Potential (MMP) Measurement

Cells were centrifuged twice and stained based on the JC‐1 MMP Detection Kit (BestBio, Shanghai, China), and the red/green fluorescence signals were captured on a flow cytometer to reflect MMP levels.

### 2.12 Reactive Oxygen Species (ROS) Detection

Cells were added with serum‐free medium diluted with DCFH‐DA (Beyotime, Shanghai, China) to reach a 10 μmol/L concentration and incubated for 20 min. A fluorescence microplate reader was applied (488 nm excitation and 525 nm emission wavelength).

### 2.13 LDH Release Assay

LDH release was quantified using a commercially available kit (Nanjing Jianjian Bioengineering Institute). Cell supernatant (120 μL) and LDH detection solution (60 μL) were mixed and reacted for 30 min. The measurement of absorbance at 490 nm was conducted with a Bio‐Rad microplate reader.

### 2.14 Detection of Antioxidant and Inflammatory Indicators

Oxidative stress parameters, superoxide dismutase (SOD), catalase (CAT) and malondialdehyde (MDA) in skeletal muscle tissue homogenates and cell supernatants, along with tumor necrosis factor‐α (TNF‐α) and interleukin‐1β (IL‐1β) were measured by ELISA kits (Nanjing Jianjian Bioengineering Institute, Nanjing, China).

### 2.15 Western Blot

The total protein was collected through radio‐immunoprecipitation assay buffer (Beyotime) and its quantity was measured using the BCA kit (Beyotime). Combined with a single sample buffer, the protein was boiled in water at 100°C for 5 min. Subsequently, 100 μg of protein were separated with 100 g/L SDS‐PAGE (Beyotime), moved to a PVDF membrane (Millipore, USA), and treated with a 50 g/L skimmed milk powder–TBST solution for 1 h. The primary antibodies Bax (1:1000, ab32503, Abcam, USA), Bcl‐2 (1:1000, ab196495, Abcam), Cleaved Caspase‐3 (1:1000, ab2302, Abcam), Keap1 (1:2000, 10,503‐2‐AP, Proteintech, Wuhan, China), Nrf2 (1:1000, 80,593‐1‐RR, Proteintech), HO‐1 (1:1000, 10,701‐1‐AP, Proteintech), and GAPDH (1:5000, ab181602, Abcam) were reacted overnight in incubation at 4°C, succeeded by a 1 h room temperature incubation with horseradish peroxidase‐tagged secondary antibody (1:3000, Proteintech). The protein bands were visualized by ECL (Pierce, USA) and analyzed using the ChemiDocTM MP system (Bio‐Rad).

### 2.16 Reverse‐Transcription Quantitative Real‐Time Polymerase Chain Reaction (RT‐qPCR)

Total RNA was extracted from tissues and cells by Trizol (Invitrogen) and qualified. The cDNA product was obtained by reverse transcription of the extracted RNA using the reverse transcription kits according to the manufacturer's instructions (Takara, Japan). Subsequently, the qPCR assay was performed using SYBR Green Mix (Takara) on a Real‐Time PCR system (7500, Applied Biosystems, ABI, USA). The reaction conditions were pre‐denaturation (95°C, 10 min) and 40 cycles of amplification (95°C, 15 s; 60°C, 30 s; 70°C, 30 s). Nrf2 expression was measured using the 2^−ΔΔCt^ method. GAPDH was used as the internal control. The primer information is shown in Table 1.

**TABLE 1:** Primer sequences used in PCR.

Genes	Primers (5′– 3′)
Nrf2	Forward: TGTAGATGACCATGAGTCGC
	Reverse: TCCTGCCAAACTTGCTCCAT
GAPDH	Forward: TCCCATCACCATCTTCCA
	Reverse: CATCACGCCACAGTTTTCC

Abbreviations: GAPDH, glyceraldehyde 3‐phosphate dehydrogenase; Nrf2, Nuclear factor‐erythroid factor 2‐related factor 2.

### 2.17 Statistical Analysis

The data were statistically analyzed using GraphPad Prism 8.0 (GraphPad Software, USA). Every piece of data was presented as mean ± standard deviation, utilizing the *t* test for contrasting the two groups and the One‐Way ANOVA along with Tukey's multiple comparisons test for group comparisons. *p <* 0.05 was considered statistically significant.

## 3 Results

### 3.1 MOAH Alleviates Skeletal Muscle I/R Injury

**FIGURE 1:**
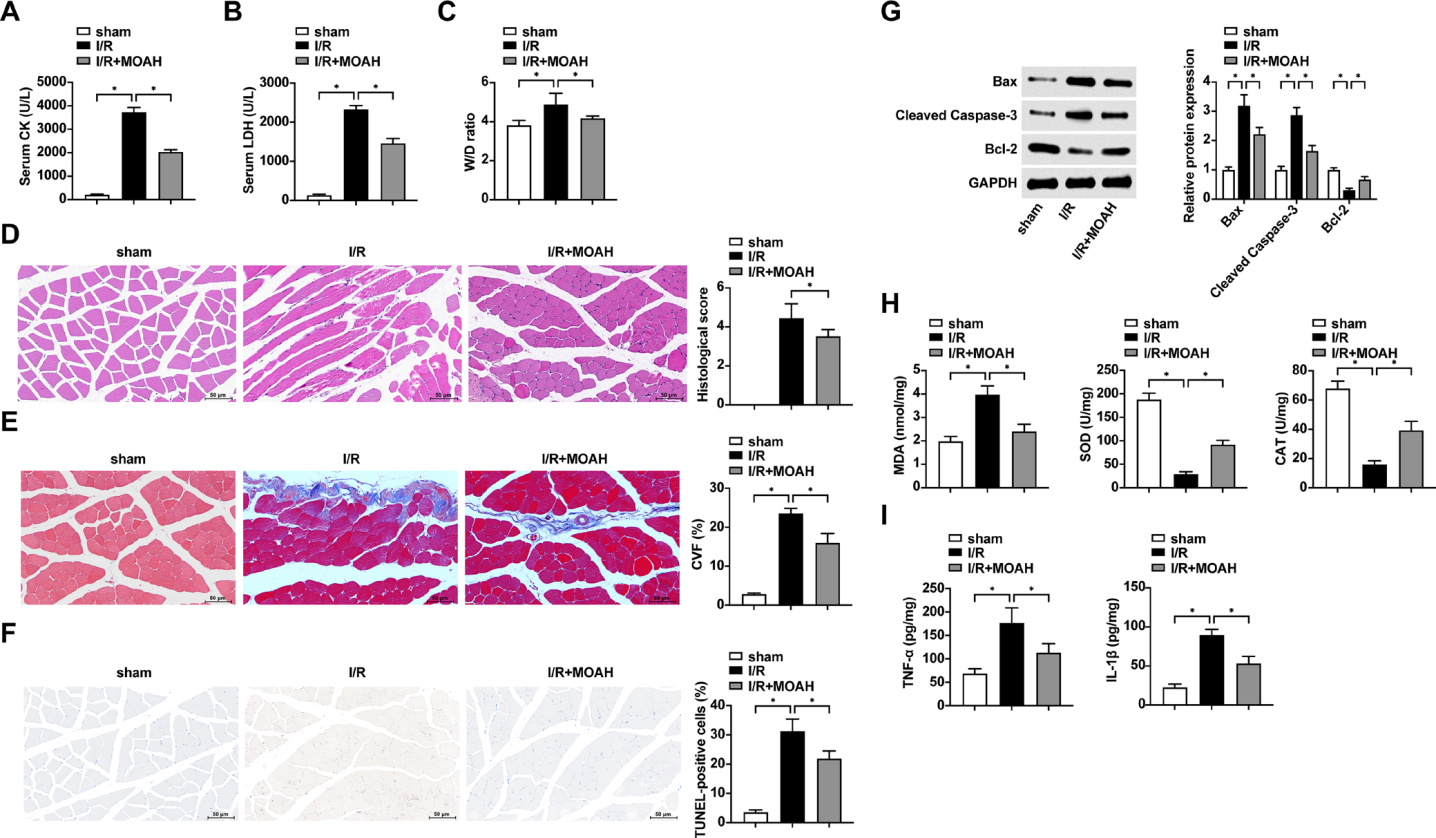
MOAH alleviates I/R‐induced skeletal muscle injury. (A) Comparison of serum CK level; (B) Comparison of serum LDH level; (C) Comparison of W/D ratio of skeletal muscle; (D) H&E staining to observe the damage of skeletal muscle tissue; (E) Masson staining to observe the area of collagen deposition in skeletal muscle tissue; (F) TUNEL staining to observe the ratio of apoptosis in skeletal muscle tissue; (G) Western blot to measure protein expression levels of Bax, Cleaved Caspase‐3 and Bcl‐2 in rat skeletal muscle tissues; (H) Comparison of SOD, CAT and MDA in rat skeletal muscle tissues; (I) Comparison of TNF‐α and IL‐1β in rat skeletal muscle tissues. *n* = 10, * indicates *p* < 0.05.

A rat model of lower limb I/R injury was established through femoral artery ligation and subsequent release. Rats undergoing I/R received pretreatment with MOAH. Biochemical analysis revealed elevated serum CK and LDH levels in I/R rats, with MOAH intervention demonstrating significant reduction of these biomarkers (Figure 1A,B). Quantitative assessment of tissue hydration via W/D ratio confirmed pronounced skeletal muscle edema in I/R animals, while substantially ameliorated by MOAH administration (Figure 1C). H&E and Masson's trichrome staining demonstrated marked tissue damage and collagen deposition in I/R rats, with MOAH treatment showing protective effects against both structural deterioration and fibrotic progression (Figure 1D,E). Apoptotic pathway analysis via TUNEL staining and Western blot revealed increased apoptotic index accompanied by elevated Bax and Cleaved Caspase‐3 expression alongside suppressed Bcl‐2 levels in the I/R group. MOAH pretreatment effectively counterregulated these apoptotic mediators (Figure 1F,G). Oxidative stress parameters analysis showed decreased SOD and CAT activity with concurrent elevation of MDA in I/R rats, while inflammatory markers TNF‐α and IL‐1β exhibited significant upregulation. MOAH administration restored antioxidant enzyme activity and reduced both lipid peroxidation and proinflammatory cytokine levels (Figure 1H,I).

### 3.2 Ozone Alleviates OGD/R‐Induced Skeletal Muscle Cell Injury

**FIGURE 2:**
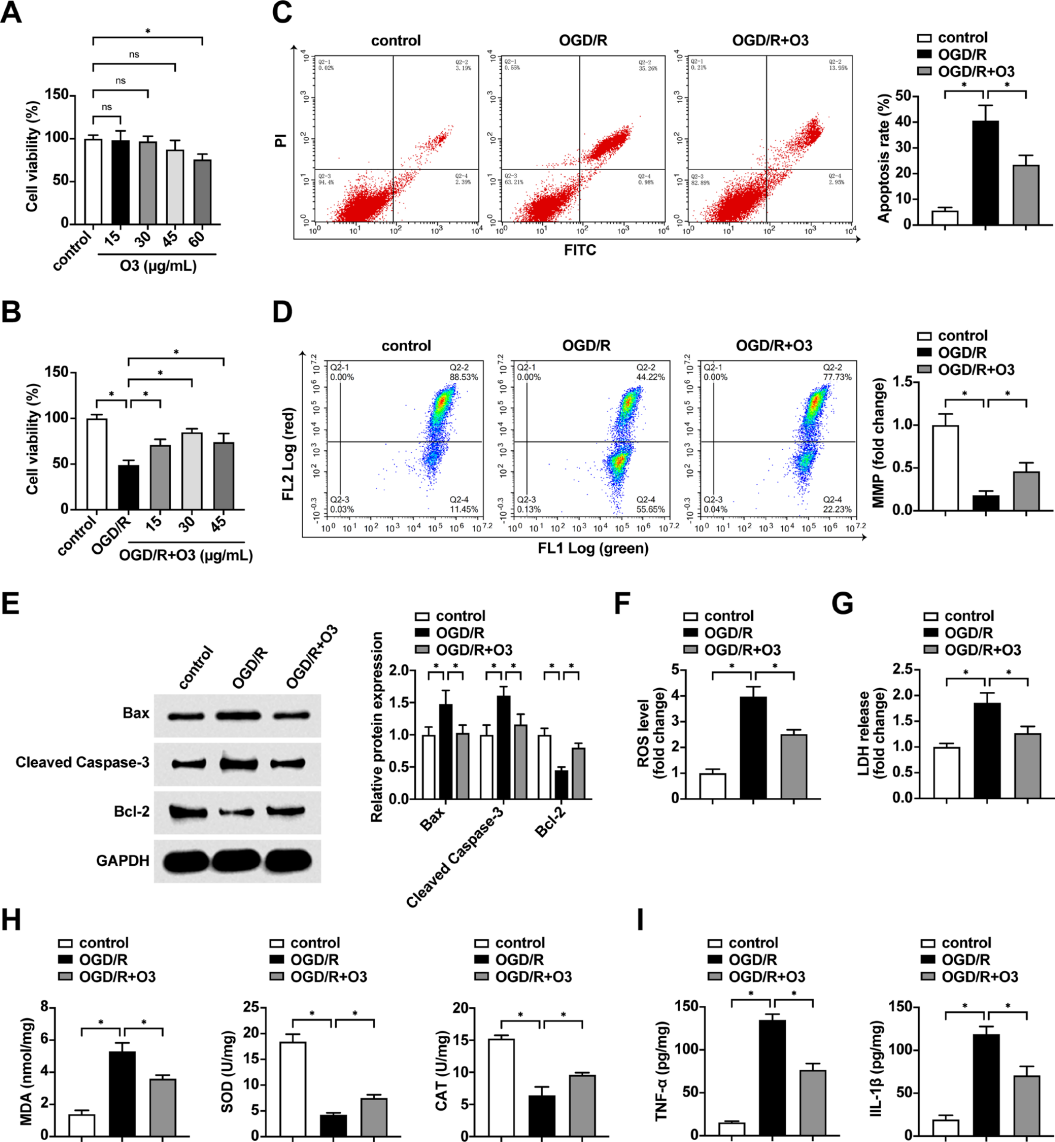
Ozone alleviates OGD/R‐induced skeletal muscle cell damage. (A) Effect of different concentrations of ozone pretreatment on the viability of normal L6 cells; (B) Effect of different concentrations of ozone pretreatment on the viability of OGD/R‐induced L6 cells; (C) Detection of apoptosis rate by flow cytometry; (D) Detection of MMP by flow cytometry; (E) Detection of the protein expression levels of Bax, Cleaved Caspase‐3, and Bcl‐2 in cells by Western blot; (F) Comparison of ROS levels in cells; (G) Comparison of LDH release rates; (H) Comparison of SOD, CAT and MDA levels in cell supernatants; (I) Comparison of TNF‐α and IL‐1β levels in cell supernatants. Experiments were independently repeated 3 times. ns indicates not significant, * indicates *p* < 0.05.

Ozone at 15, 30, and 45 μg/mL had no significant effect on the cell viability of normal skeletal muscle cells. The viability of OGD/R‐induced skeletal muscle cells was enhanced by different concentrations of ozone intervention (15, 30, and 45 μg/mL), and especially the effect of ozone pretreatment at the concentration of 30 μg/mL was the most obvious; therefore, 30 μg/mL was selected for the subsequent cell experiments (Figure 2A,B). Detection of apoptosis and cellular MMP levels by flow cytometry revealed (Figure 2C,D) that OGD/R induction led to increased apoptosis and decreased MMP, and ozone pretreatment decreased apoptosis and increased MMP. Western blot detection of apoptotic proteins showed the same trend (Figure 2E), with OGD/R induction increasing Bax and Cleaved Caspase‐3 and decreasing Bcl‐1 protein expressions. Bax and Cleaved Caspase‐3 were suppressed, and Bcl‐1 protein expression was enhanced after ozone pretreatment. OGD/R resulted in elevated cellular ROS levels and LDH release rates, and ozone pretreatment could effectively reduce the changes in these two parameters (Figure 2F,G). OGD/R induction resulted in a significant decrease in cellular SOD and CAT activity, whereas MDA, TNF‐α, and IL‐1β levels were all significantly elevated. In contrast, ozone pretreatment decreased cellular MDA, TNF‐α and IL‐1β levels and elevated cellular SOD and CAT activity (Figure 2H,I).

### 3.3 Ozone Regulates the Nrf2/HO‐1 Pathway In Vivo and In Vitro

Nrf2 regulates downstream cytoprotective genes including HO‐1 to counteract oxidative stress and apoptosis. To investigate the involvement of Nrf2 signaling in MOAH‐mediated protection against skeletal muscle I/R injury, we systematically evaluated the regulatory effects of MOAH on Nrf2, its endogenous inhibitor Keap1 (Kelch‐like ECH‐associated protein 1), and downstream effector HO‐1.

**FIGURE 3:**
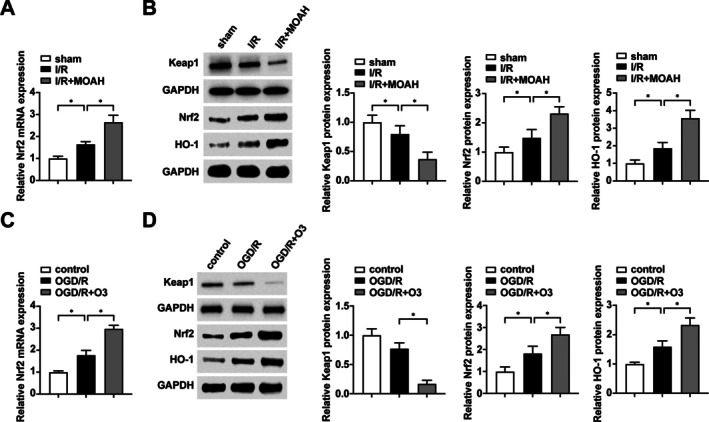
Ozone regulates the Nrf2/HO‐1 signaling pathway in vivo and in vitro. (A) RT‐qPCR to detect the expression of Nrf2 mRNA in rat skeletal muscle tissues; (B) Western blot to detect the expression of Keap1, Nrf2 and HO‐1 in rat skeletal muscle tissues; (C) RT‐qPCR to detect the expression of Nrf2 mRNA in cells; (D) Western blot to detect the expression of Keap1, Nrf2 and HO‐1 in cells. *n* = 10. Experiments were independently repeated 3 times. * indicates *p* < 0.05.

RT‐qPCR and Western blot revealed that I/R injury induced upregulation of Nrf2 and HO‐1 expression in skeletal muscle tissues, concomitant with Keap1 downregulation. MOAH treatment further enhanced this regulatory pattern, demonstrating amplified Nrf2 and HO‐1 activation coupled with stronger Keap1 suppression (Figure 3A,B). Complementary in vitro studies recapitulated these findings. OGD/R challenge increased Nrf2 and HO‐1 expression while decreasing Keap1 levels. Ozone pretreatment augmented this response, yielding more pronounced Nrf2/HO‐1 upregulation and Keap1 downregulation compared to untreated OGD/R controls (Figure 3C,D). These regulatory patterns across experimental models substantiate that Nrf2/HO‐1 pathway activation constitutes a crucial mechanism through which MOAH exerts its therapeutic effects in skeletal muscle I/R injury.

### 3.4 MOAH Ameliorates I/R‐Induced Skeletal Muscle Injury by Regulating Nrf2/HO‐1

**FIGURE 4:**
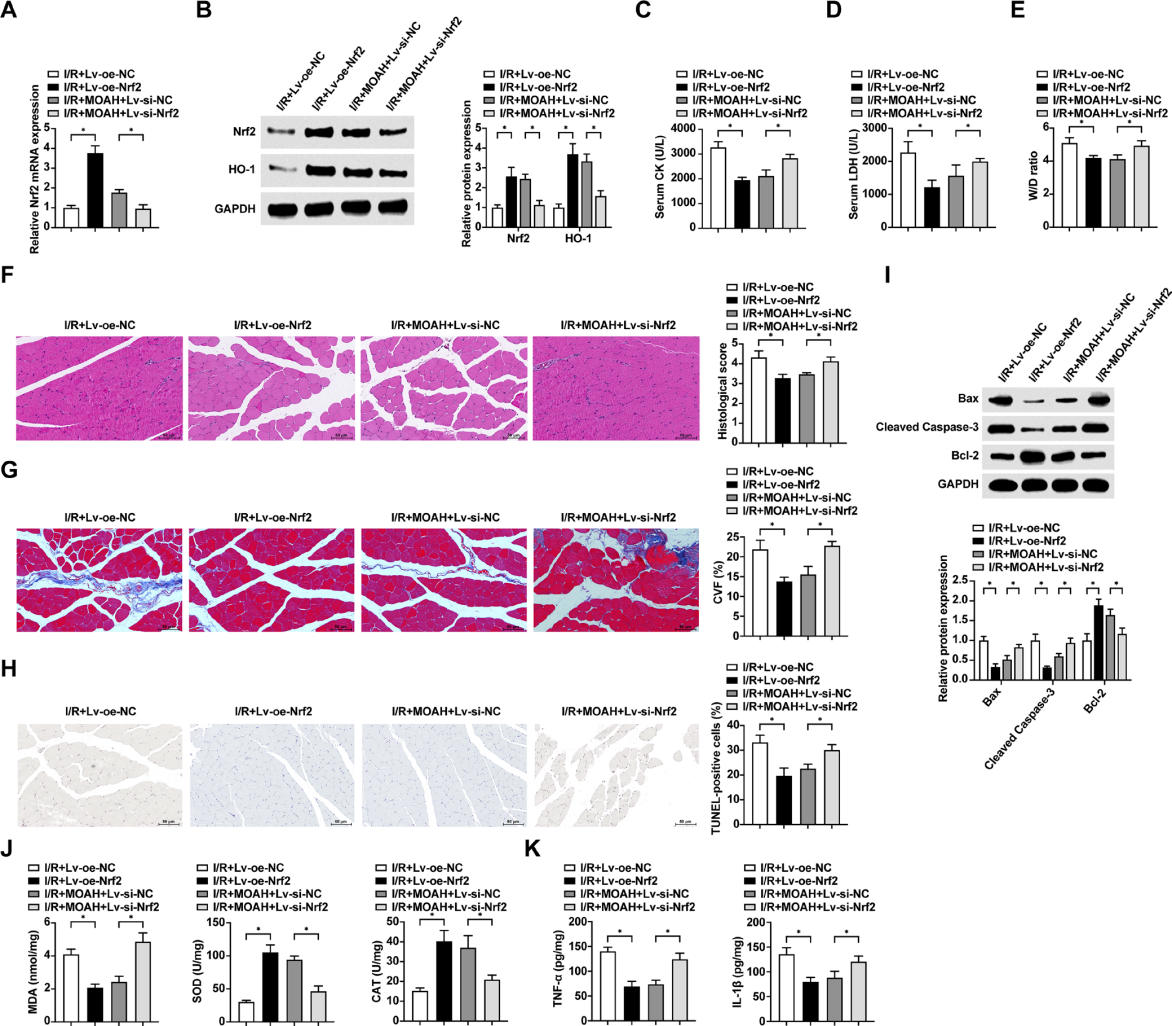
MOAH ameliorates I/R‐induced skeletal muscle injury by regulating Nrf2/HO‐1. (A) RT‐qPCR to detect the expression of Nrf2 mRNA in the skeletal muscle tissues of rats; (B) Western blot to detect the expression of Nrf2 and HO‐1 proteins in the skeletal muscle tissues of rats; (C) Comparison of serum CK levels; (D) Comparison of serum LDH levels; (E) Comparison of W/D ratio in the skeletal muscle; (F) H&E staining to observe the damage of skeletal muscle tissues in rats; (G) Masson staining to observe collagen fibers in rat skeletal muscle tissues; (H) TUNEL staining to observe the apoptosis ratio of rat skeletal muscle tissues; (I) Western blot to detect the protein expression levels of Bax, Cleaved Caspase‐3 and Bcl‐2 in rat skeletal muscle tissues; (J) Comparison of SOD, CAT and MDA levels in rat skeletal muscle tissues; (K) Comparison of TNF‐α and IL‐1β levels in rat skeletal muscle tissues. *n* = 10, * indicates *p* < 0.05.

Lentiviral treatments altered Nrf2 expression in I/R rats and expression of the Nrf2 downstream factor HO‐1 (Figure 4A,B). Nrf2 overexpression effectively suppressed I/R‐induced elevation of serum CK and LDH levels in rats, whereas Nrf2 knockdown reversed the suppression of serum CK and LDH levels by MOAH treatment (Figure 4C,D). Nrf2 overexpression alleviated edema of rat skeletal muscle tissues, whereas Nrf2 knockdown mitigated the improvement of MOAH treatment on edema in rat skeletal muscle tissues (Figure 4E). The tissue damage and collagen fibrosis were alleviated by Nrf2 overexpression, but Nrf2 silencing weakened the alleviating effect of MOAH on tissue damage and collagen fibrosis of I/R rats (Figure 4F,G). In addition, overexpression of Nrf2 reduced the apoptosis rate and Bax and Cleaved Caspase‐3 levels, whereas Nrf2 knockdown impaired MOAH‐targeted protection against apoptosis (Figure 4H,I). Nrf2 overexpression effectively decreased MDA, TNF‐α and IL‐1β levels and elevated SOD and CAT activity. Knockdown of Nrf2, on the other hand, reversed the improvement in oxidative stress and inflammatory response to MOAH treatment (Figure 4J,K).

### 3.5 Ozone Ameliorates OGD/R‐Induced Skeletal Muscle Cell Injury by Modulating Nrf2/HO‐1

**FIGURE 5:**
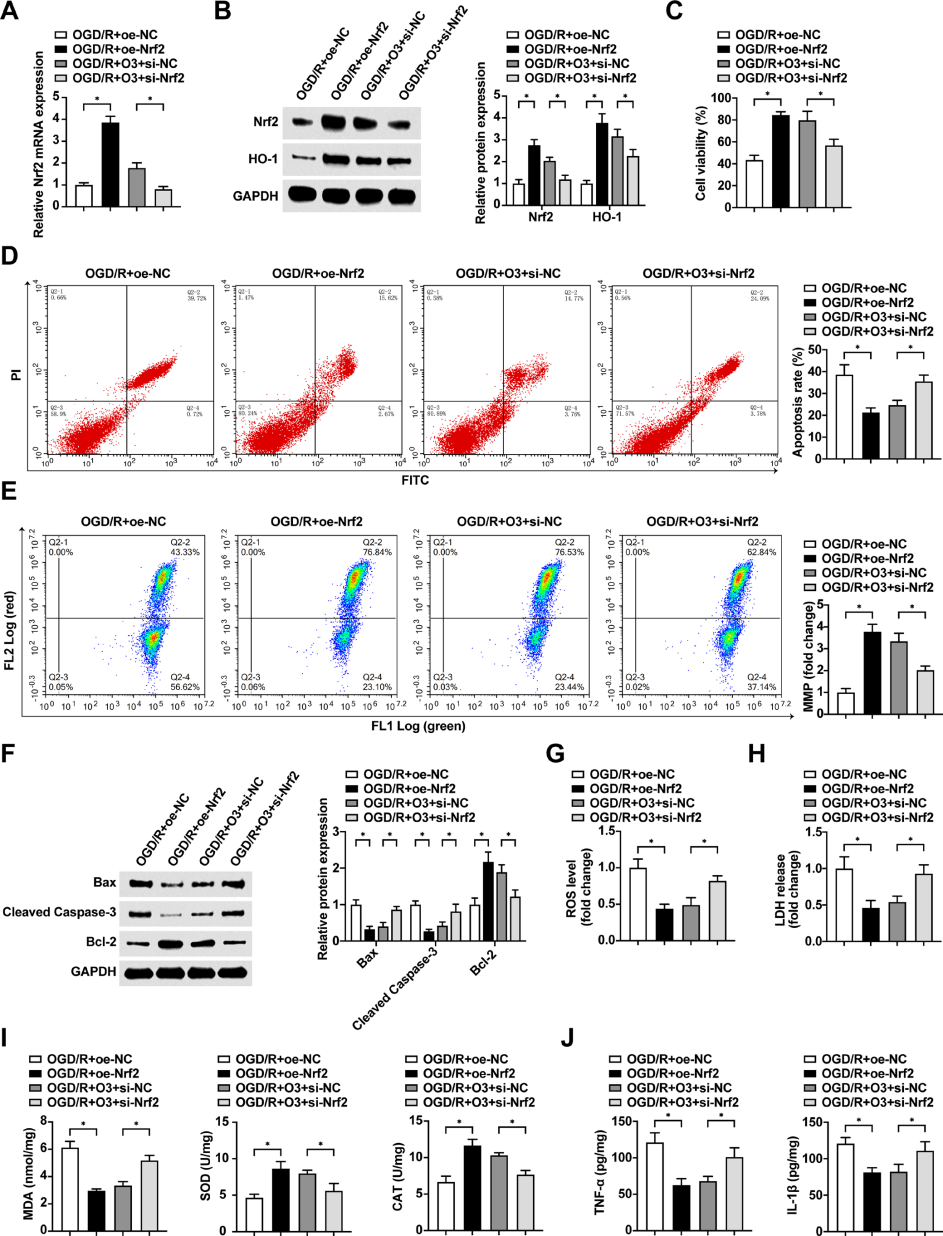
Ozone ameliorates OGD/R‐induced skeletal muscle cell injury by regulating Nrf2/HO‐1. (A) RT‐qPCR to detect Nrf2 mRNA in cells; (B) Western blot to detect Nrf2 and HO‐1 proteins in cells; (C) CCK‐8 to detect the viability of cells; (D) Flow cytometry to detect the apoptosis rate of cells; (E) Flow cytometry to detect the MMP of Cells; (F) Western blot to detect the protein expression levels of Bax, Cleaved Caspase‐3 and Bcl‐2 in cells; (G) Comparison of ROS levels in cells; (H) Comparison of LDH release rates in cells; (I) Comparison of SOD, CAT and MDA levels in cell supernatants; (J) Comparison of TNF‐α and IL‐1β levels in cell supernatants. Experiments were independently repeated 3 times, * indicates *p* < 0.05.

Cellular Nrf2 expression was altered by transfection of related sequences, and the expression of the downstream factor HO‐1 was similarly altered (Figure 5A,B). CCK8 and flow cytometry revealed (Figure 5C–E) that Nrf2 overexpression elevated cell viability and MMP and reduced apoptosis; whereas the protective benefits of ozone on cell viability and MMP, as well as its reduction of apoptosis, were reversed by silencing Nrf2. Meanwhile, Western blot indicated that Bax and Cleaved Caspase‐3 proteins were inhibited and Bcl‐1 protein expression was enhanced after overexpression of Nrf2, while suppressing impaired the action of ozone on these indicators (Figure 5F). Up‐regulating Nrf2 could effectively inhibit the increase of cellular ROS level and LDH release rate caused by OGD/R, and downregulating Nrf2 blocked the reduction of cellular ROS level and LDH release rate by ozone pretreatment (Figure 5G,H). Elevated Nrf2 significantly reduced cellular MDA, TNF‐α, and IL‐1β levels, while significantly increasing SOD and CAT activity. Down‐regulating Nrf2 reversed the alleviation of oxidative damage and inflammatory response of cells by ozone pretreatment (Figure 5I,J).

## 4 Discussion

The significance of skeletal muscle I/R injury in medical settings should not be overlooked, with well‐known pathophysiological factors including calcium overload, inflammation, oxidative stress, and mitochondrial damage [6]. When oxidative stress occurs, oxygen and its derived free radicals are extremely chemically active, attacking the cell membrane and oxidizing with its unsaturated fatty acids, disrupting the ionic permeability and integrity of cells, as well as affecting the lipid microenvironment of membrane‐bound enzymes, receptors, and ions, causing intracellular calcium overload and injury [25]. The wet‐to‐dry weight ratio of skeletal muscle, as well as levels of antioxidant indices, were elevated in the rat lower limb I/R injury model. Moreover, light microscopy observed disturbed muscle fiber alignment, inflammatory cell infiltration, and expanded collagen fiber area in I/R rats. Considering the inflammatory response is recognized as a key mechanism in skeletal muscle I/R injury [26], this study detected pro‐inflammatory factors and finally revealed that TNF‐α and IL‐1β were elevated in skeletal muscle tissues of lower limb I/R rats. Observations of serum CK and LDH levels, as well as apoptosis in skeletal muscle tissue, further reflected skeletal muscle damage after I/R induction. These conditions were alleviated by pretreatment with MOAH. In cardiac I/R, ozone treatment shows positive therapeutic effects [27]. Ozone application also attenuates I/R‐induced musculocutaneous flap injury in the pectoral muscle [28]. The present in vitro cellular experiments further validated the protective effect of ozone on skeletal muscle I/R injury. Ozone pretreatment showed effective therapeutic effects on OGD/R‐induced apoptosis, mitochondrial dysfunction, oxidative stress, and pro‐inflammatory factors in skeletal muscle cells. Not coincidentally, ozone treatment has similarly shown positive protective effects in an in vitro model of cardiomyocyte I/R [29, 30].

By means of the Nrf2/Slc7a11/Gpx4 signaling pathway, ozone guards the heart from I/R injury, and ozone treatment releases Keap1‐Nrf2 interactions and supports Nrf2's nuclear translocation, leading to increased antioxidant enzyme levels [21]. Therefore, we next investigated the effects of MOAH/ozone pretreatment on the Nrf2/HO‐1 pathway. Nrf2 is a transcription factor with a leucine zip structure that exerts transcriptional activation by recognizing and binding to sequences in the genome known as antioxidant response elements. In physiological settings, Nrf2 is sequestered in the cytoplasm through binding to its cytosolic repressor Keap1, which facilitates its continuous degradation via the ubiquitin‐proteasome system, thereby preventing the translocation of Nrf2 to the nucleus, where it would have signaling effects. In response to cellular damage, Nrf2 dissociates from Keap1 and translocates to the nucleus, where it binds antioxidant response elements to promote the expression of genes that protect cells, such as HO‐1 [31, 32]. HO‐1 is known for its protective function, as it transforms heme into carbon monoxide (CO), free iron, and biliverdin, providing anti‐inflammatory, anti‐apoptotic, and antioxidant effects. One of the key protective systems of cells against environmental damage is the Nrf2/HO‐1 pathway [33, 34]. By activating this pathway, cells might be safeguarded from oxidative stress, apoptosis, and inflammation linked to I/R injury [35, 36]. Upregulation of HO‐1 enhances antioxidant enzyme activity, including SOD and CAT, which neutralize ROS and reduce lipid peroxidation products such as MDA, thereby mitigating oxidative stress and inflammation [34]. Activation of Nrf2/HO‐1 pathway promotes Bcl‐2/Bax heterodimerization, preventing Bax oligomerization and subsequent mitochondrial outer membrane permeabilization. This inhibition suppresses the release of apoptosis‐inducing factor (AIF) and cytochrome c (Cyt c), thereby preventing the activation of initiator caspase (caspase‐9) and subsequently blocking the activation of executioner caspase (caspase‐3). Consequently, this process preserves DNA integrity from AIF‐induced damage and mitigates cellular apoptosis [37].

**FIGURE 6:**
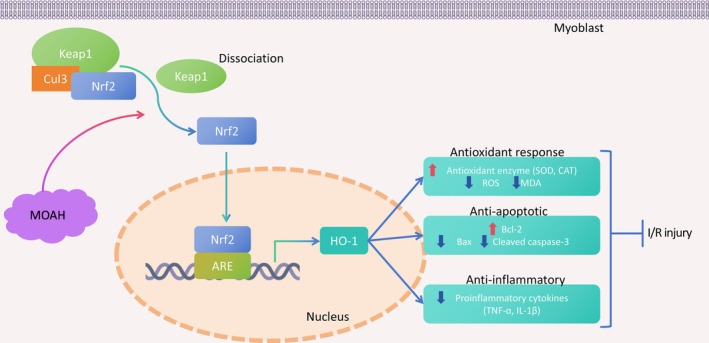
A graphical abstract of the mechanism by which MOAH meliorates skeletal muscle I/R injury.

The Nrf2/HO‐1 pathway serves as a protective mechanism against I/R injury across multiple organ systems, including the brain [38] and heart [39]. Its pharmacological activation via agents such as lipoxin A4 and syringic acid has previously been shown to mitigate I/R‐induced skeletal muscle damage and apoptosis [40], a protective paradigm further corroborated by our experimental results. In the current investigation, MOAH/ozone preconditioning robustly activated the Nrf2/HO‐1 axis, as evidenced by significant upregulation of both Nrf2 and HO‐1 in I/R‐injured skeletal muscle tissues and OGD/R‐induced myoblasts. Crucially, genetic suppression of Nrf2 abolished the protective effects of MOAH in vivo, while similarly neutralizing ozone's cytoprotective capacity in vitro. This demonstrated rescue of therapeutic efficacy upon Nrf2 suppression confirms pathway specificity, establishing MOAH‐mediated Nrf2/HO‐1 activation as a central therapeutic mechanism in skeletal muscle I/R injury management. The MOAH intervention facilitates Keap1 degradation, liberating Nrf2 for nuclear translocation, where it transcriptionally activates HO‐1 expression. This coordinated signaling cascade effectively attenuates the pathophysiological triad of I/R injury—oxidative stress through enhanced free radical scavenging, apoptotic cell death via mitochondrial pathway regulation, and inflammatory response suppression via cytokine modulation (Figure 6).

The present study acknowledges two principal methodological limitations. First, the intrinsic technical challenges associated with replicating MOAH intervention in cell culture systems limit our ability to fully elucidate the therapeutic mechanisms of MOAH in vitro. Second, although our experimental design included standard I/R controls, future studies would benefit from incorporating an additional I/R control group that receives ozone‐free autohemotherapy to better isolate and validate MOAH‐specific therapeutic mechanisms. Notwithstanding these considerations, the current findings provide compelling evidence that Nrf2/HO‐1 pathway modulation constitutes the principal mechanism underlying the protective effects of MOAH against skeletal muscle I/R injury.

## 5 Conclusions

Our study reveals that MOAH has great potential value for the management of skeletal muscle I/R injury. In addition, our findings underscore the indispensable role of Nrf2/HO‐1 signaling in mediating the therapeutic benefits of MOAH against skeletal muscle I/R injury, positioning it as a central mechanistic target for clinical translation. However, the clinical translatability of MOAH requires further substantiation through rigorously controlled clinical trials, and complementary preclinical investigation remains essential.

## Ethics Statement

The animal experiment research protocol was approved by the Ethics Committee of The First Affiliated Hospital of Jinan University (PY‐LL‐2024‐0128) and performed in accordance with the “Guidelines for the care and use of experimental animals.”

## Conflicts of Interest

The authors declare no conflicts of interest.

## Data Availability

The datasets used and/or analyzed during the present study are available from the corresponding author on reasonable request.
